# Characteristics and Long-Term Outcome of Cerebellar Strokes in a Single Health Care Facility in Mexico

**DOI:** 10.7759/cureus.28993

**Published:** 2022-09-09

**Authors:** Rodolfo Villalobos-Díaz, Laura A Ortiz-Llamas, Luis A Rodríguez-Hernández, José G Flores-Vázquez, Metztli Calva-González, Marcos V Sangrador-Deitos, Michel G Mondragón-Soto, Rodrigo Uribe-Pacheco, Eliezer Villanueva Castro, Manuel A Barrera-Tello

**Affiliations:** 1 Neurosurgery, Instituto Nacional de Neurología y Neurocirugía Manuel Velasco Suárez, Mexico City, MEX; 2 Neurosurgery, Hospital Regional Mérida, Instituto de Seguridad y Servicios Sociales de los Trabajadores del Estado (ISSSTE), Mérida, MEX; 3 General Surgery, Centro Medico American British Cowdray (ABC), Mexico City, MEX

**Keywords:** cerebellar stroke, obstructive hydrocephalus, decompressive craniectomy, cerebellum, posterior cranial fossa

## Abstract

Objective

The purpose of this study was to analyze and discuss the clinical characteristics, long-term outcome, and prognostic factors of cerebellar strokes treated in a single health care facility in Mexico.

Methods

We retrospectively reviewed the medical records of adult patients admitted to our hospital with diagnosis of cerebellar ischemic and hemorrhagic stroke between 2018 and 2020. Baseline data included sociodemographic and radiological variables, treatment (surgical versus conservative), and Glasgow Coma Scale on arrival (GCSOA). The final neurological outcome was evaluated with the Glasgow Outcome Scale (GOS) six months after hospital discharge.

Results

Ten patients (seven male and three female) with a mean age of 57.9 ± 9.3 years were included, six with cerebellar ischemic infarction and four with cerebellar hemorrhage. Out of the 10 patients, four underwent surgery (suboccipital decompressive craniectomy {SDC} ± ventriculostomy). The outcome was favorable in four cases (40%) and unfavorable in six (60%). Patients who underwent surgical treatment fared worse with all four cases associating poor outcome. The comparison between good and poor outcome groups showed significant differences in the presence of obstructive hydrocephalus (one versus six, p = 0.05) and poorer GCSOA (6.16 ± 1.72 versus 12.5 ± 3.6, p = 0.05), associating poorer outcome.

Conclusion

There is still controversy regarding the appropriate management of cerebellar strokes. The presence of obstructive hydrocephalus and poorer GCSOA are associated to worse outcomes.

## Introduction

Cerebellar stroke is an often-misdiagnosed clinical condition that is potentially worsened by serious complications such as edema and brain stem compression. The cerebellum is composed of a central part, the vermis, and two lateral expansions, the hemispheres that, along with the brain stem, are situated in the posterior cranial fossa [1,2]. The critical structures contained within the posterior fossa combined with its minimal compliance lead to significant morbidity from any space-occupying lesions [3].

Cerebellar ischemic infarctions account for 2%-3% of all ischemic strokes; they occur most frequently in the fifth through the eighth decades of life and with a greater frequency in males [4,5]. Similar to ischemic strokes in the anterior circulation, the main causes of cerebellar infarction are cardioembolism and large vessel atherosclerotic disease [1,2,6]. In ischemic cerebellar infarctions, brain swelling results from both cytotoxic and vasogenic edemas, which sometimes give rise to brain stem and/or fourth ventricle compression due to the mass effect caused by the infarct and the consequent surrounding edema [7]. On the other hand, cerebellar hemorrhages account for approximately 9%-10% of all intracranial hemorrhages and are more frequently seen in males and in middle-aged to older patients (usually beyond the fifth decade) [8]. These hemorrhages are mainly associated with vascular changes secondary to hypertension (60%-90% of cases); coagulopathies, aneurysms, neoplasms, and amyloid angiopathy account for the remainder [7,8]. In these patients, the causes of deterioration are variable and include increased mass effect from surrounding edema or expansion of the hematoma from repeated bleeding. Either mechanism can cause direct brain stem compression or obstructive hydrocephalus [8,9].

The mainstay of surgical treatment for massive cerebellar strokes is the suboccipital decompressive craniectomy (SDC); however, the paucity of high-quality data supporting this approach has given rise to controversy and no general agreement about the best therapeutic option [10,11]. In our country, there is scarce data regarding sociodemographic factors, treatment success rate, and long-term outcomes of this group of neurological patients.

The purpose of this study was to analyze and discuss the clinical characteristics, long-term outcome, and prognostic factors observed in 10 cases of acute cerebellar stroke treated in a single health care facility in Mexico, between June 2018 and June 2020.

## Materials and methods

Patients who were admitted to the regional hospital “Elvia Carrillo Puerto, ISSSTE” for cerebellar stroke, between June 2018 and June 2020, were eligible for inclusion in this observational and retrospective study. The inclusion criteria were age older than 18 at the time of the stroke, adequate radiological documentation of the lesion by means of computed tomography (CT), and adequate data available on the institutional electronic medical record, including sociodemographic data, comorbidities, treatment received (surgical versus conservative), and Glasgow Coma Scale on arrival (GCSOA). The final neurological outcome was evaluated in the medical records of the neurology/neurosurgery outpatient clinic after six months of hospital discharge and expressed according to the Glasgow Outcome Scale (GOS). The final outcome was dichotomized in “good outcome” for those with a GOS of 5 (good recovery) and 4 (moderate recovery) and “poor outcome” for those with a GOS of 3 (severe disability), 2 (persistent vegetative state), and 1 (death). The present study was conducted under the ethical aspects considered in the World Medical Association Declaration of Helsinki.

On the initial CT, we evaluated the size and location of the lesion, the status of basal cisterns (normal or compressed), the status of the fourth ventricle (normal or compressed), the presence of obstructive hydrocephalus, and the presence of associated lesions. The volume of the lesions was assessed by using the formula A*B*C/2, where A represents the maximum transverse diameter of the lesion, B the anteroposterior diameter, and C the number of slices (1 mm) showing the lesion.

Basic demographic data (gender, etiology, and age) and clinical and radiological parameters were analyzed to assess differences in neurological outcome (GOS). The Mann-Whitney U test was used to compare categorical variables (GOS) with continuous variables (age and lesion size on CT). Fisher’s exact test was used for comparisons between categorical variables due to the small sample size. A p value of <0.05 was considered as statistically significant. All statistical analyses were performed using Statistical Package for the Social Sciences version 17.0 (SPSS Inc, Chicago, IL, USA).

## Results

A total of 12 patients were admitted in our hospital for cerebellar stroke during the study period; however, two patients did not fulfill the inclusion criteria, leaving a total of 10 patients. Seven patients were male, while three were female. Their age of onset ranged from 45 to 71 years with an average of 57.9 ± 9.3 years. Out of the 10 cases, six were diagnosed with cerebellar infarction, while four were diagnosed with cerebellar hemorrhage, the latter being exclusive in males. The most frequent comorbidities in our population were hypertension (100%), diabetes mellitus (60%), and previous stroke (20%). The most affected vascular territory in both ischemic and hemorrhagic stroke was the posteroinferior cerebellar artery (PICA) territory with five cases (83%) and two cases (50%), respectively. There was a considerable heterogeneity within the sizes of the lesions, with an average of 50 ± 20.5 cm^3^ for the ischemic lesions and 26.9 ± 11.7 cm^3^ for those of hemorrhagic nature. Of the 10 patients who attended our facility, six were managed conservatively, and four underwent surgery, with cerebellar infarcts being the cause of emergent decompression in 3/4 (75%) cases and cerebellar hemorrhage in 1/4 (25%) cases. Among the patients surgically treated, all four patients had poor outcomes six months after discharge (two cases with severe disability and two cases with mortality). Regarding the conservatively treated group (six cases), four cases resulted in good outcome (moderate disability) and two cases in poor outcome (mortality in two cases). The conservative management included restrictive hypertonic solutions, head positioning, blood pressure and glucose, and hyperventilation through mechanical assistance if needed. The characteristics of the patients are shown in Table 1.

**Table 1:** Characteristic of 10 patients with diagnosis of cerebellar stroke M: male; F: female; Lt: left; Rt: right; Bil: bilateral; PICA: posterior inferior cerebellar artery; AICA: anterior inferior cerebellar artery; SCA: superior cerebellar artery; WSCA: watershed area in deep white; GCSOA: Glasgow Coma Scale on arrival; P: present; A: absent; C: compressed; N: normal; HT: hypertension; DM: diabetes mellitus; CI: cerebral infarction; COPD: chronic obstructive pulmonary disease; CHF: congestive heart failure; SDC: suboccipital decompressive craniectomy; GOS: Glasgow Outcome Scale; MD: moderate disability; D: death; SD: severe disability

Case	Age (years)	Sex	Type	Location by vascular territory	GCSOA	Volume (cm^3^)	Hydrocephalus (P/A)	BS (C/N)	Comorbidities	Intervention	GOS
1	58	F	Ischemic	Rt PICA	7	32.6	P	C	DM and HT	Conservative	MD
2	59	F	Ischemic	Rt PICA	3	42.7	P	C	HT	Conservative	D
3	71	M	Hemorrhagic	Rt SCA and Rt PICA	7	35.7	P	C	DM, HT, and CI	SDC + ventriculostomy	SD
4	52	M	Ischemic	Lt PICA	6	50.9	P	C	DM and HT	SDC + ventriculostomy	D
5	65	M	Hemorrhagic	Lt SCA	14	13.3	A	N	HT	Conservative	MD
6	51	M	Ischemic	Bil PICA and Rt SCA	6	103.2	P	C	DM and HT	SDC + ventriculostomy	D
7	45	M	Hemorrhagic	WSCA	15	9.4	A	N	HT and CI	Conservative	MD
8	67	M	Hemorrhagic	WSCA	8	23.6	P	N	HT, DM, COPD, and CHF	Conservative	D
9	66	M	Ischemic	Lt SCA	14	35.2	A	N	DM and HT	Conservative	MD
10	45	F	Ischemic	Rt PICA	7	35.4	P	C	HT	SDC + ventriculostomy	SD

When compared, the good and poor outcome groups showed no significant differences in age, gender, type of lesion, state of basal cisterns, or treatment received, even if there was a tendency in favor of the conservatively treated group (p = 0.07). Significant differences were found for the presence of obstructive hydrocephalus (six versus one, p = 0.05) and poorer GCSOA (6.16 ± 1.72 versus 12.5 ± 3.6, p = 0.05), both associated with poorer outcomes (Table 2).

**Table 2:** Comparison of patients with good and poor outcome for each factor GOS: Glasgow Outcome Scale; GCSOA: Glasgow Coma Scale on arrival; NSS: not statistically significant; n: number *Fisher’s exact test; **Mann-Whitney U test

Factor	GOS > 3	GOS ≤ 3	p
Cases	4	6	
Age (years)	59 ± 8.7	57.5 ± 10	NSS**
Male sex (n)	3	4	NSS*
Ischemic infarction (n)	2	4	NSS*
Basal cisterns compression (n)	1	3	NSS*
GCSOA (n)	12.5 ± 3.6	6.16 ± 1.72	<0.05**
Hydrocephalus (n)	1	6	<0.05*
Surgical treatment (n)	0	4	NSS*

## Discussion

In this clinical series, cerebellar strokes were more prevalent among men (70%), the adult population (median: 58.5 years), and those with cardiovascular risk factors, such as hypertension (100%) and type 2 diabetes (60%), which is consistent with the current literature [2,6,7,8,9]. The high prevalence of hypertension present in our study population (all cases) was remarkable, reflecting its importance as a predisposing factor for both intracerebral bleeding and atherosclerotic thrombus formation.

The analysis of factors associating poor outcome showed statistical significance for the presence of hydrocephalus on initial CT (p < 0.05) and a lower initial Glasgow Coma Scale (GCS) (p < 0.05). Several previous studies, which provided analysis of predictors for clinical outcome, agree with our findings [2,3,12-14]. In a study, St Louis et al. [15] showed the following radiological features as strong predictors for poor outcome: (a) hematoma > 3 cm in diameter, (b) brain stem compression, (c) hydrocephalus, and (d) intraventricular extension from cerebellar hemorrhage. Moreover, the German-Austrian Space-Occupying Cerebellar Infarction Study (GASCIS), a study with a prospective multicenter design, found the level of consciousness as the only predictor for poor outcome [16].

In our study, significant differences were not found in the long-term outcome or mortality between the patients with cerebellar hemorrhage and those with cerebellar infarct, as has been suggested in some studies, which report an association between cerebellar hemorrhage and poorer outcomes in comparison with cerebellar infarcts; however, our cohort requires a larger sample to draw stronger conclusions [7,13,17]. The reason for this lack of differences is probably due to the several limitations present in our study, such as the small size of the population included and its retrospective nature. A suggested explanation for the more sinister sequelae of cerebellar hemorrhage is that the continued bleeding after the start of a hemorrhage exerts a greater mass effect on the surrounding brain tissue and causes a more marked evolution of obstructive hydrocephalus (from the compression of the fourth ventricle and cerebral aqueduct) [13].

Since the first reported cases of surgery for cerebellar stroke in 1956, posterior fossa decompression has become the main surgical treatment for large cerebellar hemorrhages or cerebellar infarcts with swelling leading to brain stem compression [17,18]. However, there is currently no randomized or otherwise controlled study assessing the best treatment option in patients with cerebellar strokes; furthermore, existing publications are biased because of their small size and retrospective nature, the absence of control group, and the lack of uniform reporting of outcomes [10,11,19-21]. This has led to a profound and still active controversy regarding the appropriate management of cerebellar infarcts and hemorrhage, as well as the optimal surgical technique. The current American Heart Association/American Stroke Association (AHA/ASA) guidelines recommend SDC with dural expansion for patients who deteriorate clinically despite medical management. A ventriculostomy to relieve obstructive hydrocephalus should be accompanied by an SDC [22]. For cerebellar hematomas, AHA/ASA recommends surgical removal of hematomas more than 3 cm in diameter, in patients who deteriorate neurologically or in patients who have brain stem compression and/or hydrocephalus from ventricular obstruction [23]. However, there is no high-quality evidence to confirm or refute these recommendations. In a systematic review and meta-analysis by Kuramatsu et al., which included four observational studies and 6,580 patients with cerebellar hemorrhage, it reported that surgical hematoma evacuation (by means of SDC, craniotomy, or minimal invasive surgery) compared with conservative treatment was not significantly associated with likelihood of better functional disability at three months (30.9% versus 35.5%, p = 0.43) but was significantly associated with greater probability of survival at three months (78.3% versus 61.2%, p = 0.005) [11]. Another systematic review by Ayling et al., assessing the impact of SDC in functional outcomes and mortality in patients with cerebellar ischemic stroke, reported a 28% (95% CI, 20%-37%) rate for moderate-severe neurological disability and 20% (95% CI, 12%-31%) for mortality [10].

In our study, patients who underwent surgical treatment (SDC ± ventriculostomy) fared worse than those who received a conservative treatment, with mortality being present in two out of four cases treated surgically and poor neurological outcome (GOS) in those who survived. However, statistically significative differences between both treatment groups were not found, even if there was a tendency favoring the conservative treatment. These outcomes may be partially explained because patients with initial Glasgow score and those with larger lesions or basal cistern compression on initial CT were more likely to receive a surgical treatment than those without these characteristics. The abovementioned should be taken with caution due to the treatment indication biases of withheld surgery in some patients with good initial neurological outcome.

Cerebellar strokes have been found to result in a high morbidity and mortality, and the results of our study support this, in which poor outcome was present in 60% of the overall cases, with 40% of them resulting in mortality [8,17,24]. In the medical literature, there is a considerable heterogeneity regarding the rates for cerebellar strokes; in our study, mortality was comparable to previous case series, in which mortality reached 14%-56% [8,17,25]. Neugebauer et al. studied 115 publications on space-occupying cerebellar stroke, which included 764 patients, showing an overall mortality of 27.9% [24].

Limitations of this study include its retrospective nature, the small number of patients included from a single facility and the heterogeneity in cerebellar lesion sizes, initial neurological status, and the etiology of the stroke, which make the data difficult to compare. These limitations have been constantly mentioned in the medical literature assessing cerebellar strokes, which are majorly limited to case series and observational studies [10,11]. The latter is understandable because withholding a life-saving surgical intervention for a randomized controlled trial (RCT) will raise major ethical concerns. Therefore, a future RCT comparing surgical with conservative management in this pathology is unlikely, making the observational studies the only source of information to analyze and improve the management in cerebellar strokes.

Nevertheless, we propose to adopt the treatment recommendations suggested by the latest AHA/ASA guidelines, especially in Latin American countries, where there is a lack of epidemiological and observational studies assessing this pathology, its management, and long-term outcomes [22]. In addition, the impact of the cerebellar strokes and their treatment modalities should be assessed in a multicenter study within our population, which may show differences from the literature due to sociodemographic variations, comorbidity prevalence, and social beliefs within our population, as well as differences in our health care system.

## Conclusions

In conclusion, our study describes the outcome and long-term prognosis of 10 patients with cerebellar stroke. Despite the lack of difference between both groups in terms of treatment, it was clear that the presence of obstructive hydrocephalus and poorer GCSOA were associated with worse outcomes, which could serve as prognostic factors and help in the treatment decision making. There is still controversy regarding the appropriate management of cerebellar strokes due to the lack of high-quality evidence. We want to encourage the proper registration and publication of cerebellar stroke case series, especially in Latin American countries, where there is a lack of epidemiological and observational studies assessing this pathology, its management, and long-term outcomes.
